# Paralytic Shellfish Toxins in Coastal Waters of Changdao Island (China): Toxin Profiles, Potential Producers, and Environmental Conditions

**DOI:** 10.3390/md23050217

**Published:** 2025-05-21

**Authors:** Guanchao Zheng, Yuxiang Deng, Haiyan Wu, Xiaokang Li, Ling Cheng, Chengxu Yuan, Minlu Liu, Zhijun Tan

**Affiliations:** 1 Key Laboratory of Testing and Evaluation for Aquatic Product Safety and Quality, Ministry of Agriculture and Rural Affairs, Yellow Sea Fisheries Research Institute, Chinese Academy of Fishery Sciences, Qingdao 266071, China; 2 Shandong Key Laboratory of Marine Ecological Restoration, Shandong Marine Resources and Environment Research Institute, Yantai 264006, China; 3 Marine Economy Promotion Center, Marine Ecological Civilization Comprehensive Experimental Area of Changdao, Yantai 265800, China; 4 Third Institute of Oceanography, Ministry of Natural Resources, Xiamen 361005, China; 5 State Key Laboratory of Mariculture Biobreeding and Sustainable Goods, Yellow Sea Fisheries Research Institute, Chinese Academy of Fishery Sciences, Qingdao 266071, China

**Keywords:** paralytic shellfish toxins, contaminated shellfish, potential toxin sources, environmental parameters, Changdao Island

## Abstract

In recent decades, there have been frequent occurrences of paralytic shellfish toxin (PST) contamination in the Yellow and Bohai Seas, China. The waters around Changdao Island, situated at the convergence of these two seas, have suffered harmful algal blooms of *Alexandrium* spp., indicating a potential risk of PST contamination in shellfish. However, a systematic investigation and assessment of PSTs in this area is still lacking. The presence of PSTs in plankton concentrates and shellfish in coastal areas of Changdao Island was monitored from April to October 2022, using liquid chromatography–tandem mass spectrometry. The potential toxin-producing microalgae were analyzed, as were the environmental conditions associated with their occurrence. The highest levels of PSTs in plankton concentrates and shellfish were both observed in September, reaching levels of 105.8 ng STXeq./L and 114.7 μg STXeq./kg, respectively. The main analogues were C1, C2, and GTX1–4. High-throughput analysis of the plankton concentrates identified eight species of *Alexandrium*, which are potential producers of PSTs. Sediment samples also revealed the presence of permanent cysts of *Alexandrium*. This research represents a significant advance in our understanding of the distribution and hypothetical sources of PSTs in the coastal waters of Changdao Island.

## 1. Introduction

Harmful algal blooms (HABs) have impacted coastal regions worldwide, leading to significant ecological and economic damage [1,2]. The shellfish toxins found in HABs are metabolic products released by specific microalgae, such as cyanobacteria and dinoflagellates [3,4]. Among these toxins, paralytic shellfish toxins (PSTs) are the most extensively distributed and are extremely potent marine biotoxins [5]. Shellfish primarily accumulate PSTs in their bodies through filter-feeding on toxin-producing algae. The accumulated PSTs are then transferred up the food chain to human consumers [6]. Each year, the global consumption of shellfish contaminated with PSTs results in over 2000 human poisoning incidents, posing a significant threat to human health and severely affecting marine ecological safety, and has resulted in global concern [7,8]. PSTs are a group of potent neurotoxic alkaloids including saxitoxin (STX) and at least 57 of its derivatives, mainly produced by eukaryotic dinoflagellates in the genera *Alexandrium*, *Gymnodinium,* and *Pyrodinium* [9,10]. PSTs can be divided into five major classes based on the differences in their R4 groups: carbamates, including GTX1-4, STX, NEO, and some M-toxins; N-sulfocarbamoyl, including GTX5, GTX6, C1-4, and some M-toxins; decarbamoyl, including dcSTX, dcNEO, and dcGTX1-4; deoxydecarbamoyl, including doSTX, doGTX1, and doGTX2; and benzoates, including the newly discovered GC-toxins [11,12]. PSTs can induce muscle paralysis, respiratory distress, and in extreme instances, death in humans [13,14]. PSTs inhibit the transmission of neuronal impulses in peripheral nerves and skeletal muscles by reversibly blocking voltage-gated Na^+^ channels [15]. Research suggests that the toxicity of the various analogues of PSTs may be related to the binding of the R4 group to receptor site 1 of the Na^+^ channel [16,17]. In order to better protect consumer health, the European Food Safety Authority (EFSA) has set a safety limit of 800 μg STXeq./kg [18]. The World Health Organization, the Food and Agriculture Organization of the United Nations, the United States, Canada, etc., have also adopted this limit [19,20,21].

PST toxin-producing phytoplankton species, mainly *Alexandrium* and *Gymnodinium*, are widely distributed along the Chinese coast [22]. Studies carried out in Chinese waters have reported that *A. andersonii*, *A. leei*, and *A. catenella* occur in the Chinese Yellow Sea (YS) and Bohai Sea (BS), whereas *A. tamarense* has been detected in the South China Sea [23]. Similarly, *A. andersonii* and *A. leei* have been found in Jiaozhou Bay, also situated in the YS and BS [24]. The main source of PSTs in the YS and BS are dinoflagellate species of the genus *Alexandrium*, and the toxins detected include C1, C2, GTX1–4, dcGTX2 and dcGTX3, etc. [25,26]. PSTs are also prevalent in shellfish from coastal waters of the BS, with PST toxicities often exceeding the 800 μg STXeq./kg safety limit, leading to numerous poisoning events with serious socio-economic impacts on human health and the fisheries industry [27,28]. PST contamination levels found in the vicinity of the YS far exceeded the safety limits, with the highest levels of PSTs found in scallops, reaching 3953.5 µg STXeq./kg, followed by clams, with 993.4 µg STXeq./kg [29].

Located at the convergence of the YS and BS, Changdao Island in Yantai City, Shandong Province, may be at risk of PST contamination. In 2006, a bloom of PST-producing *Alexandrium* spp. was reported as the causative agent of a mass mortality of caged fish in the waters off Changdao [30]. A survey of red tide events caused by *A. tamarense*, a producer of PSTs, was carried out in the waters around Nanhuangcheng Island in Changdao County, with a total affected area of 2.37 km^2^. Additionally, PST-producing *Gymnodinium* spp. were detected in coastal waters of Yantai’s Sishili Bay and in Weihai City (near Changdao Island), with an affected area of 48.88 km^2^ [31]. Changdao is an important scallop farming area in Shandong Province. Shandong Province produced 1,023,104 tons of scallops in 2023, accounting for approximately 55.17% of China’s total scallop production [32], but research on PSTs in the coastal waters of Changdao Island remains limited.

There has been very little comprehensive and systematic research on PSTs in coastal waters of Changdao Island, especially regarding the types and concentrations of the different PSTs, and the characteristics and potential sources of PST contamination have not yet been clarified [33,34]. In this study, shellfish, water samples, and sediment samples were collected from designated locations in coastal waters of Changdao Island, and PST analyses were carried out using liquid chromatography–tandem mass spectrometry (LC-MS/MS). The contamination status of PSTs was clarified by comparing the temporal changes in toxin concentrations and composition, and the distribution characteristics of the various toxins. High-throughput sequencing and optical microscopy observations enabled us to trace the hypothetical sources of PSTs, the toxin-producing alga involved, and the environmental conditions associated with their occurrence. This study is of great significance to the sustainable development of the fisheries industry around Changdao Island and to the protection of consumers’ health, and it also increases our understanding of the shellfish contamination status in this area.

## 2. Results

### 2.1. Toxin Profiles and Concentrations of PSTs in Plankton Filters

**Figure 1:**
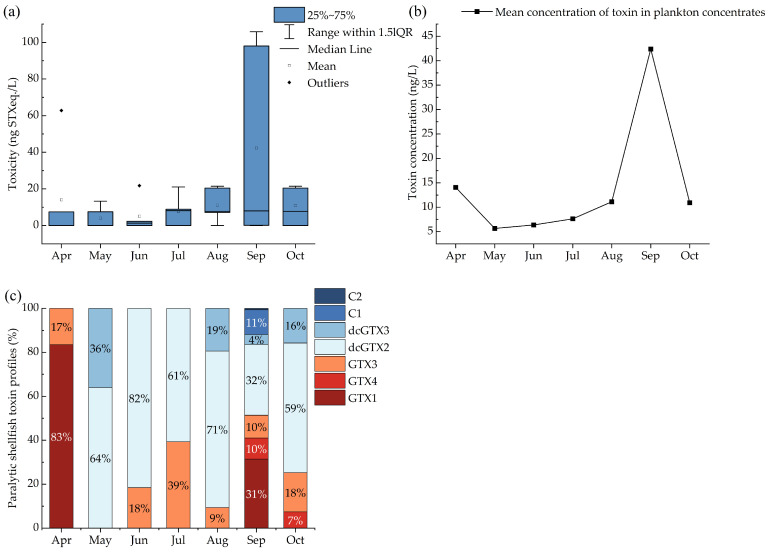
PST (**a**) toxicity, (**b**) mean concentration, and (**c**) profiles in plankton concentrates in water samples by month.

Of the 41 water samples   that were collected from April to October 2022 for analyses of toxins in plankton concentrates, 23 contained detectable levels of PSTs, resulting in a detection rate of 56.1%. The detected toxicity ranged from 7.1 to 105.8 ng STXeq./L (Figure 1a), with the lowest PST levels observed in May and the highest in September. We calculated the mean toxin concentration in plankton concentrates from different sites in the same month to describe their seasonal occurrence (Figure 1b). The highest PST levels were found in September. The mean concentration values from April to October ranged from 4.0 to 42.4 ng/L. 

Figure 1c shows the toxin profile with the contribution of the different PSTs in the plankton concentrates. A total of seven PST analogues were detected, including C1, C2, dcGTX2, dcGTX3, GTX1, GTX4, and GTX3. Differences in the occurrence of PST compounds were observed across the different months. Fewer PST analogues were detected from April to July, namely, dcGTX2, GTX3, GTX1, and dcGTX3. The diversity of PST analogues increased from August onwards, with the greatest variety occurring in September, when seven analogues were identified. This month also saw a higher proportion of GTX1, which exhibited the highest toxicity among the analogues discovered (Figure 1a). Analogues C1 and C2 were only detected in September, each comprising less than 15% of the total PST content.

### 2.2. Seasonal Distribution of PSTs in Shellfish Samples

**Figure 2:**
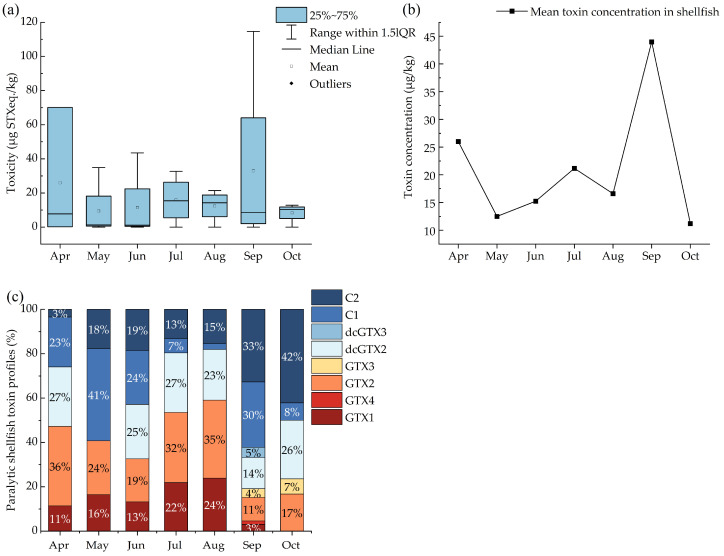
Seasonal distribution of PSTs in shellfish: (**a**) toxicity, (**b**) mean concentration, and (**c**) profiles in shellfish samples by month.

A total of 21 shellfish samples were collected throughout the survey period, with PSTs detected in 17 of these, resulting in a detection rate of 81.0%. The mean PST toxicity from April to October ranged from 7.1 to 44.0 μg STXeq./kg, with the lowest mean PST toxicity observed in May. Toxicity levels showed an upward trend from May to September, reaching a peak in September (Figure 2a), closely corresponding to the rising trend observed in the plankton concentrates. We calculated the mean toxin concentration in shellfish from different sites in the same month to describe monthly trends (Figure 2b). The September values were the highest. Among the various shellfish samples, specimens of *Crassostrea gigas* collected in May had the lowest PST toxicity, with only 0.2 μg STXeq./kg, while the highest toxicity was found in *Chlamys farreri* specimens sampled in September, which reached 114.7 μg STXeq./kg.

A total of eight PST compounds were detected in the shellfish extracts, which closely resembled the PST compounds found in plankton concentrates, including C1, C2, dcGTX2, dcGTX3, GTX1, GTX4, GTX2, and GTX3 (Figure 2c). The distribution of PST compounds across the different months was similar, although the proportion of these compounds differed compared with those in the plankton concentrates. The less toxic compounds, C1, C2, and GTX2, were detected every month and constituted a high proportion (up to 84%) of the total toxin content. In contrast, the more toxic GTX1 was not detected in October, and GTX3 was only detected in small amounts in September and October (constituting 4% and 7% of the total toxin content, respectively). September exhibited the highest diversity of compounds, with all eight compounds detected, whereas May had the lowest diversity, with only four compounds identified. Overall, the trends in PST toxicity in the plankton concentrates and shellfish samples were generally consistent in the different months. Although similar PST compounds were detected in both types of samples throughout the survey, there were differences in the composition and relative proportions of PSTs in the same month. Notably, GTX2 was not detected in the plankton concentrates, whereas it was detected in the shellfish samples. These findings suggest that further research is necessary to elucidate the mechanisms underlying the enzymatic transformation of the toxins in shellfish.

### 2.3. Preliminary Identification of the PST-Producing Algal Genera

**Figure 3:**
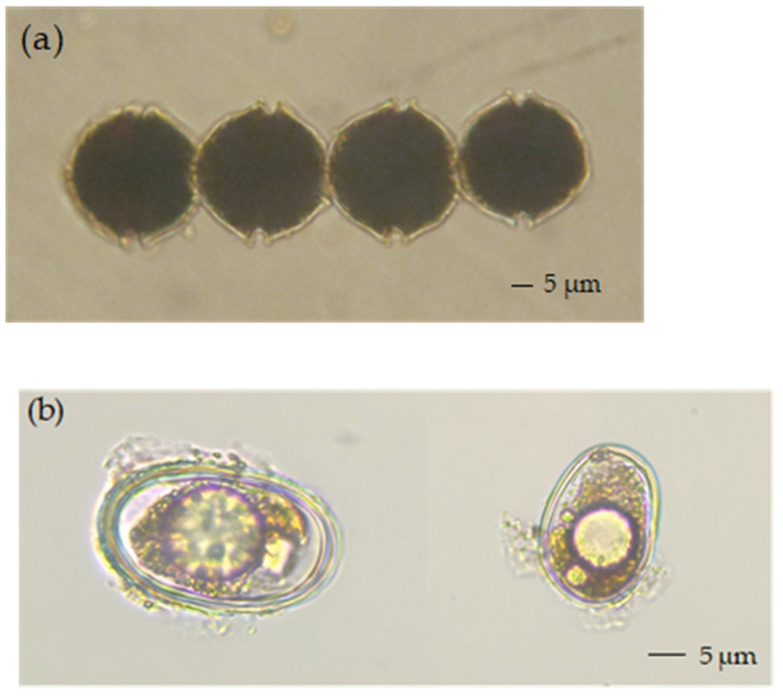
Some cells (**a**) and cysts (**b**) of *Alexandrium* spp. observed in the samples.

**Figure 4:**
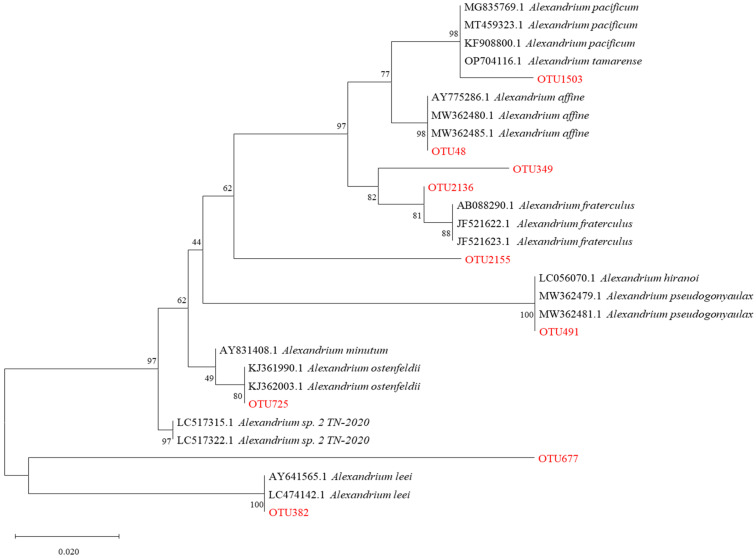
Phylogenetic tree established for selected *Alexandrium* spp. and the nine OTUs assigned to them based on the 18S rDNA V4 region.

Microscopic examination of the Lugol-fixed water samples revealed cells of *Alexandrium* spp. (Figure 3a). Similarly, examination of the sediment samples revealed cysts of *Alexandrium* spp. (Figure 3b). Despite these observations, the exact genera and species of the PST-producing algae in the coastal waters of Changdao Island could not be accurately determined. A total of 550 operational taxonomic units (OTUs) were assigned to eukaryotic microalgae, based on the high-throughput sequencing results. Following a previously published method [9] utilizing the NCBI database BLASTing results, a total of nine OTUs were identified as belonging to *Alexandrium* spp. (Table 1). The OTU48, OTU491, OTU1503, and OTU382/OTU677 sequences, previously assigned to *A. affine*, *A. hiranoi*/*pseudogonyaulax*, *A. pacificum*/*tamarense*, and *A. leei*, respectively, showed high identity (98–100%) with the corresponding *Alexandrium* spp., while the OTU2136, OTU349, OTU2155, and OTU725 sequences, previously assigned to *A. fraterculus* and *A. ostenfeldii*, respectively, showed lower identities (92–95%). Nonetheless, a phylogenetic tree constructed using the 18S rDNA V4 region sequences (maximum likelihood method) still supported their assignments (Figure 4). *Alexandrium hiranoi*/*pseudogonyaulax* and *A. pacificum*/*tamarense* were consistently detected from July to October; *A. affine* was observed in May, June, and July; *A. ostenfeldii* persisted from June to October; *A. leei* was detected only in September; and *A. fraterculus* was observed in September and October.

### 2.4. Monthly Variations in Environmental Parameters

**Figure 5:**
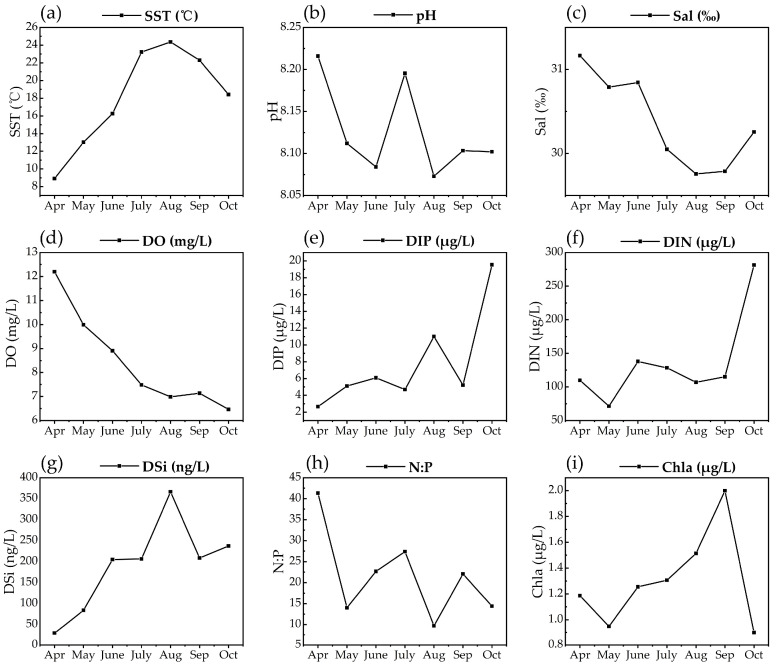
Monthly variations in environmental parameters observed in the coastal waters of Changdao Island. (**a**) Sea surface temperature (°C), (**b**) pH, (**c**) salinity (‰), (**d**) DO (mg/L), (**e**) DIP (μg/L), (**f**) DIN (μg/L), (**g**) DSi (ng/L), (**h**) N:P, (**i**) chlorophyll a (μg/L).

Sea surface temperature (SST) (Figure 5a) and inorganic nutrient levels exhibited marked changes during the survey period. The SST increased from April to August, reaching a maximum of 24.36 °C in August, followed by a decline from September to October. Throughout the survey period, there was no discernible trend in pH variation over time, with values fluctuating slightly within the range of 8.1 to 8.2 (Figure 5b). Salinity exhibited a relatively steady decline from 31.17 to 29.76 (Figure 5c). The most pronounced change observed was the decrease in dissolved oxygen (DO) content, with the highest level (12.20 mg/L) recorded in April. Subsequently, DO levels declined continuously, reaching their lowest point in October (6.47 mg/L), and they only rebounded slightly to 7.14 mg/L in September (Figure 5d). The dissolved inorganic phosphate (DIP) concentration fluctuated within a relatively stable range from April to September, showing a slight increase or decrease of approximately 2 μg/L each month (Figure 5e). However, in October, the DIP level surged suddenly to a peak concentration of 19.54 μg/L, with a large increase compared to other months (Figure 5e). Similar to the trend observed for DIP, the dissolved inorganic nitrogen (DIN) concentration fluctuated generally, with slight increases before October, ranging from 71.44 to 137.98 μg/L, before rising to 281.36 μg/L in October (Figure 5f). The dissolved silicon (DSi) concentration showed a quick rise before August and then began to fall in September (Figure 5g). The nitrogen/phosphorus (N:P) ratio did not display any distinct pattern of change, showing an overall fluctuating downward trend. The highest value (41.30) was recorded in April, and the lowest value (9.72) in August (Figure 5h). The chlorophyll a maximum was reached in September before rapidly declining to its lowest level in October (Figure 5i).

## 3. Discussion

Due to the frequent occurrence of HABs in the YS and BS in recent years, researchers have studied the contamination status of PSTs in these areas [34]. However, there is limited understanding of PSTs in the plankton concentrates and shellfish, as well as the potential toxin-producing microalgal species occurring in coastal waters around Changdao Island. This study analyzed the PSTs found in plankton concentrates and shellfish samples collected in the coastal waters of Changdao Island from April to October 2022. Among all samples analyzed, maximal toxicities in plankton concentrates (105.8 ng STXeq./L) and shellfish samples (114.7 µg STXeq./kg) were found in September. The concentration of PSTs in *Alexandrium* spp. in the northern YS was examined, and PSTs were detected in most of the samples, with concentrations ranging from 0 to 11.73 pmol/L [35]. Earlier studies detected PSTs in phytoplankton in the YS, with a maximum concentration of 45.3 pmol/L [25]. These studies indicated a widespread risk of contamination arising from the PSTs produced by *Alexandrium* spp. in the YS and BS, including the Changdao Island area sampled in our study. In an earlier study of PSP toxicity in shellfish, the highest PST toxicity (546.09 µg STX eq./kg) was detected in the northern YS [36]. In a previous work, PST levels found in shellfish from the northern BS ranged from 62 to 394 μg STX eq./kg, which are values close to those found in our study [37]. Dalian, also in the YS and BS regions, had a 94.4% prevalence of PSTs in scallop and clam samples during an autumn contamination survey, with a maximum toxicity of 3953.5 µg STX.2HCl eq./kg for scallops and 993.4 µg STX.2HCl eq./kg for clams, and both values were well above the safety limit [29].

**Figure 6:**
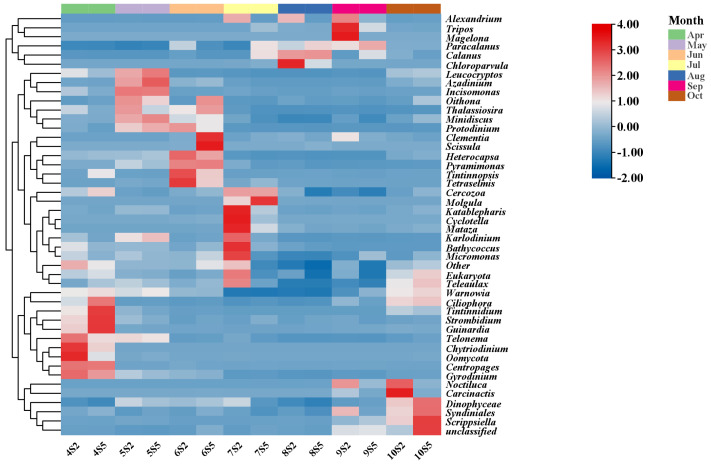
Heatmap of phytoplankton relative abundance based on high-throughput sequencing.

Overall, PST levels found in the coastal waters of Changdao Island in this study are not very serious. Nevertheless, the YS and BS are areas where PST contamination is frequent. Consequently, continuous monitoring of PSTs around Changdao Island is required. To identify the source of PSTs in the coastal waters of Changdao Island, microscopic examination and high-throughput sequencing of plankton concentrates were performed. Our results showed that *Alexandrium* spp. occurred from April to October, with the highest relative abundance observed in September (Figure 6), the same month in which the observed phytoplankton toxin profiles reached their peak. Furthermore, cysts of *Alexandrium* spp. were detected in the sediment samples (Figure 3b). These results suggest that the toxin-producing microalgal species in the coastal waters of Changdao Island belong to the genus *Alexandrium*.

Dinophycean dinoflagellates are very abundant in coastal waters of the YS and BS sea areas [25,29,35,38], and several studies have analyzed the potential PST-producing microalgae in these regions. Four *Alexandrium* spp. that produce PSTs were reported from the Liaodong Peninsula, located in the northern part of the BS, namely *A. affine*, *A. fraterculus*, *A. leei*, and *A. pseudogonyaulax* [39]. These algae corresponded to OTU48, OTU349/2155/2136, OTU382/677, and OTU491, found in the high-throughput sequencing analysis of our study (Table 1). Another study, utilizing qPCR detection methods combined with microscopic observation, identified *A. fundyense* as the primary PST-producing algae in the northern part of the YS, while *A. pacificum*, also a PST producer, was found between the YS and the East China Sea, near the Yangtze River estuary [40]. Eight known species of *Alexandrium* in the Qingdao Jiaozhou Bay area of the YS have been successfully identified, namely *A. affine*, *A. andersonii*, *A. catenella*, *A. leei*, *A. minutum*, *A. ostenfeldii*, *A. pacificum*, and *A. pohangense*, and the abundance of *A. affine* reached its peak in September, indicating that temperature may be a crucial driver of the temporal dynamics of these *Alexandrium* species [41]. The above studies show that PSTs are usually associated with *Alexandrium* spp. in the YS and BS, which corroborates findings of this study about the occurrence of potential PST-producing microalgae in coastal waters off Changdao Island.

Different *Alexandrium* spp. may produce different PST analogues. Researchers have isolated and cultured *A. affine* and *A. leei*, which have been found to produce minute quantities of GTX1, GTX4, GTX2, and GTX3 [42]. In addition, *A. tamiyavanichi* produces GTX1/4 and GTX2/3, while *A. pacificum* produces C1/2, dcGTX2/3, GTX1/4, GTX2/3, neoSTX, and STX, and other toxins have also been found [34]. In this study, the PST analogues detected in the plankton concentrates and shellfish samples mainly included GTX1/4, GTX2/3, C1/2, and dcGTX2/3. Combined with the PST analogues detected, the results of high-throughput sequencing, cyst microscopy, and previous research have shown that the PST-producing algae in the coastal waters of Changdao Island may belong to the genus *Alexandrium*, including *A. hiranoi*/*pseudogonyaulax*, *A. pacificum*/*tamarense*, and *A. leei*. In addition, PSTs were detected in all of the shellfish samples, with scallops having the most toxic PSTs, with GTX1/4, GTX2/3, and STX being the main analogues [20]. In shellfish, a certain degree of interconversion may occur between the different PST analogues, usually from the less toxic N-sulfocarbamoyl toxin to the more toxic carbamate toxin [43]. This bioconversion usually occurs in the digestive glands of shellfish [44], which can convert the less stable β-epimers (C2, GTX3, GTX4) into the more stable α-epimers (C1, GTX1, GTX2) [45]. In our study, the composition of PSTs in the plankton concentrates differed from that found in shellfish, probably due to the bioconversion of β-epimers to α-epimers in shellfish, such as the conversion of GTX3 to GTX2.

Previous surveys have shown that the abundance of *Alexandrium* spp. was closely related to SST [46]. In laboratory experiments, the growth of *A. pacificum* under different temperatures has been tested. The highest density and PST toxicity of *A. pacificum* were found at 20 °C [47]. Another study of *A. affine* cultured at different temperatures showed that the highest cell density (6724 cells/mL) and growth rate (0.22 days^−1^) were observed at 23–27 °C and that cell density was temperature-dependent in all treatments [48]. When the production of PSTs by *A. pacificum* (CCMA-272) isolated from the East China Sea was measured at different temperatures (15, 20, and 25 °C), results showed that the toxicity production at values 20 and 25 °C was comparable. However, as the temperature increased, *A. pacificum* released a greater number of highly toxic analogs (such as STX and dcGTX2) into the environment than those released intracellularly [49]. Another study showed that the toxicity of PSTs was highest in the BS plankton concentrates collected in September and that seasonal changes in the characteristics of phytoplankton PSTs may correspond to the occurrence of red tides in the BS [26]. Another study carried out in the YS and BS region showed that temperature is a crucial factor influencing the abundance of Dinophyceae. The higher temperatures in summer (23.97 ± 1.39 °C) and autumn (20.49 ± 0.30 °C) in this region promote the growth of Dinophyceae compared with spring and winter [50]. Seasonal changes in temperature lead to seasonal Dinophyceae dynamics, which are closely related to climate warming, as revealed in earlier studies [51].

A study investigating the effect of *A. catenella* on DO in coastal waters found that its growth and reproduction resulted in a significant decrease in DO levels [52]. The growth of *Alexandrium* spp. may lead to a decrease in DO levels. However, no significant effects of DO concentrations on cyst germination rates were detected in studies of *A. pacificum* cultured at different DO concentrations [53] or in studies of *A. fundyense* and *A. pacificum* [54]. In addition to SST affecting the growth rate and toxicity of *Alexandrium* spp., inorganic nutrients such as DIN and DIP are also important abiotic factors [55]. Experimental results revealed that N deficiency reduced cell growth and toxin production, while P deficiency decreased cell growth but increased toxin production [56]. Another study showed a negative correlation between the DIN concentration and the abundance of dinoflagellates [50].

HABs can cause serious ecological problems and harmful effects on the biota [2]. For example, there may be severe declines in DO, dramatic changes in N/P ratios, and deterioration in water quality in nearby waters. These can lead to marine mammal mortality, red tides, fish kills, and outbreaks of shellfish poisoning [57]. In order to combat the damage caused by HABs, data related to the sampling sites in the coastal waters of Changdao Island require continuous monitoring to better understand the potential drivers of algal growth and HAB outbreaks of *Alexandrium* spp. and to study the impacts of environmental changes on their growth and toxin production.

## 4. Materials and Methods

### 4.1. Study Area

**Figure 7:**
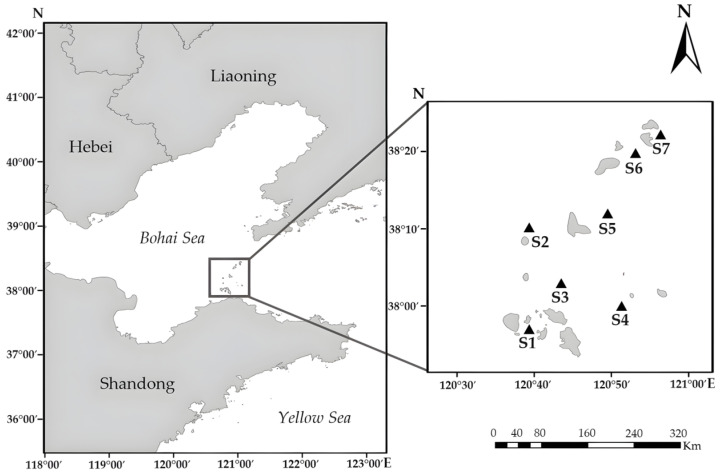
Study area and locations of the sampling sites for the collection of water and sediment samples in the coastal waters around Changdao Island.

Water samples for analysis of particulate toxins were collected in the Changdao Sea area of Yantai City, Shandong Province at stations S1 (120.39° E, 37.95° N); S2 (120.38° E, 38.10° N); S3 (120.42° E, 38.05° N); S4 (120.52° E, 38.02° N); S5 (120.48° E, 38.13° N); S6 (120.88° E, 38.22° N); and S7 (120.92° E, 38.28° N) (Figure 7). The hydrological and meteorological conditions in Yantai’s Changdao Island area were derived from the National Meteorological Science Data Center (http://data.cma.cn/site/index.html, accessed on 20 November 2024), supplemented by field observations (Table S1).

### 4.2. Field Sample Collection, Storage, and Analysis

Samples were collected monthly from April through October 2022. The water environmental sample collection method was as follows: 500 mL of seawater was vacuum-filtered through a GF/C filter (1.2 μm), and the filtered water was transferred into a clean plastic bottle and frozen at −20 °C for preservation and the subsequent detection of DIN, DIP, etc. After thawing the samples at 4 °C, the nutrients, such as NO_2_-N, NO_3_-N, NH_4_-N, DIP, DSi, and chlorophyll a, were measured using the spectrophotometric method [58]. Oceanographic parameters were recorded directly (ProPlus, YSI, Yellow Springs, OH, USA), including the salinity, temperature, DO content, and pH of seawater [27].

Sampling protocols for the plankton samples are described in Lin et al. [59]. In short, 5 L of seawater was sieved through a 200 μm mesh to remove micro-zooplanktonic organisms, and then the filtered water was passed through GF/C filters (1.2 μm) and PC filters (0.22 μm, Whatman, Chalfont St. Giles, UK). The plankton concentrates from four GF/C filters were used for the LC-MS/MS analysis of PSTs, while those from the PC filter were used for qPCR analyses. These samples were immediately frozen at −20 °C and later at −80 °C for storage. Additionally, 1 L of seawater was filtered through a mesh size of 20 μm, the filtered material was resuspended in 100 mL of filtered seawater, and then the sample was fixed with 0.4% Lugol’s solution for morphological identifications with the aforementioned inverted microscope. The plankton taxa were identified at the species level or the lowest feasible taxonomic level [58]. Sediment samples were collected at S2 and S5, kept in the dark at 4 °C, and analyzed within 2 weeks. Cysts were extracted from sediment samples using a density gradient of sodium polytungstate with a density of 1.4 g/cm^3^ and observed morphologically using an Eclipse TS100 microscope (Nikon, Tokyo, Japan) [60].

Monthly shellfish samples were collected from the coastal aquaculture farms near sites S2, S3, and S5 around Changdao Island (Figure 7). Sampled shellfish species included *C. gigas*, *C. farreri*, and *Mytilus galloprovincialis*. Each sample of *C. farreri* and *M. galloprovincialis* weighed 2 kg, but that of *C. gigas* weighed 3 kg. All shellfish samples were frozen at −20 °C for preservation and then promptly transferred and frozen at −80 °C. All samples collected from April to October were processed together to eliminate inter-batch variation.

In this study, we used high-throughput sequencing data obtained from plankton concentrates collected at stations S2 and S5 to identify the PST-producing phytoplankton species present in coastal waters around Changdao Island. Protocols for sample collection, preparation, and analyses followed those described in Cheng et al. [61]. The V4 hypervariable region of 18S rDNA was chosen as the target for high-throughput sequencing analysis, as per previous studies [62,63]. The region was amplified using a pair of universal primers for eukaryotes: forward primer D514 (5′-TCCAGCTCCAATAGCGTA-3′) and reverse primer B706R (5′-AATCCRAGAATTTCACCTCT-3′). The reaction was set up as follows: microbial DNA(10 ng/µL) 2 µL; amplicon PCR forward primer (10 µM) 1 µL; amplicon PCR reverse primer (10 µM) 1 µL; 2× Hieff^®^ Robust PCR Master Mix (Yeasen, 10105ES03, Shanghai China) (to make 30 µL in total). The plate was sealed and the PCR was performed in an Applied Biosystems 9700 (Applied Biosystems, Waltham, MA, USA) using the following program: 1 cycle of denaturing at 95 °C for 3 min, the first 5 cycles of denaturing at 95 °C for 30 s, annealing at 45 °C for 30 s, elongation at 72 °C for 30 s, then 20 cycles of denaturing at 95 °C for 30 s, annealing at 55 °C for 30 s, elongation at 72 °C for 30 s, and a final extension at 72 °C for 5 min. The PCR products were checked using electrophoresis in 2% (*w*/*v*) agarose gels in TBE buffer (Tris, boric acid, EDTA), stained with ethidium bromide and visualized under UV light. Hieff NGS™ DNA Selection Beads (Yeasen, 10105ES03, Shanghai China) were used to purify the free primers and primer dimer species in the amplicon product. Samples were sent to Sangon BioTech (Shanghai, China) for library construction using a universal Illumina adaptor and index. Before sequencing, the DNA concentration of each PCR product was determined using a Qubit^®^ 4.0 Green double-stranded DNA assay (Thermo Scientific, Waltham, MA, USA) and quality controlled using a bioanalyzer (Agilent 2100, Santa Clara, CA, USA). Depending on coverage needs, all libraries could be pooled for a single run. The amplicons from each reaction mixture were pooled in equimolar ratios based on their concentration. Sequencing was performed using an Illumina MiSeq system (Illumina Inc., San Diego, CA, USA), according to the manufacturer’s instructions.

After sequencing, the two short Illumina readings were assembled using PEAR software (v. 0.9.8) according to the overlap, and fastq files were processed to generate individual fasta and qual files, which were then analyzed using the standard methods. The effective tags were clustered into OTUs of ≥97% similarity using Usearch software (v. 11.0.667). Chimeric sequences and singleton OTUs (with only one read) were removed, after which the remaining sequences were sorted based on their OTUs. The tag sequence with the highest abundance was selected as a representative sequence within each cluster. Bacterial and fungal OTU representative sequences were classified taxonomically by blasting against the RDP and NCBI databases (https://blast.ncbi.nlm.nih.gov, accessed on 20 November 2024), respectively. Referring to the protocol [64], a total of 30 sequences of the 18S rDNA V4 region were downloaded from GenBank for species in the genus *Alexandrium*. These sequences, along with those of the nine OTUs assigned to *Alexandrium* spp. in our study, were utilized to construct a maximum-likelihood tree using the variance estimation method in Mega 11 (https://megasoftware.net/, accessed on 20 November 2024) with bootstrap replications of 10,000, and the 3-parameter + G nucleotide substitution model [65]. MrBayes v. 3.2.6 was then used to construct a Bayesian tree with 1,000,000 generations, a sampling frequency of 100, and the HKY + F + G4 nucleotide substitution model selected using Modelfinder [63].

### 4.3. Sample Processing for Toxin Extraction and Analyses

Chromatographic-grade formic acid (≥98.0%) and ammonium formate (≥97.0%) were purchased from Fluka Chemie GmbH (Buchs, Switzerland). Chromatography-grade ammonia and mass spectrometry-grade acetic acid were provided by Sigma-Aldrich (Shanghai, China). Mass spectrometry-grade acetonitrile was supplied by the Merck Company (Rahway, NJ, USA). A graphitized carbon black solid phase extraction column (ENVI-Carb™, 250 mg/3 mL) was obtained from Supelco (Bellefonte, PA, USA). PST standards (STX, dcSTX, NEO, dcNEO, GTX1&GTX4, GTX2&GTX3, GTX5, dcGTX2&dcGTX3, and C1&C2) were provided by the National Research Council of Canada (Halifax, NS, Canada). The water used (18.2 MΩcm) was deionized using a Milli-Q system (Millipore, Bedford, MA, USA) equipped with ion exchange and carbon filters.

Plankton concentrates were extracted as described in Li et al. [37]. In short, a GF/C filter membrane was divided into smaller pieces and placed in a 15 mL centrifuge tube, which was extracted using 5 mL of 1% aqueous acetic acid solution. An ultrasonic cell crusher (JY96-II, Scientz Biotechnology Co., Ltd., Ningbo, China) was set at 100 W, and the algal samples were pulverized in an ice bath. This process involved crushing the samples for 5 s and allowing them to stand for 2 s, with this procedure repeated continuously for 10 min. Microscopic examination confirmed the complete fragmentation of the algal cells, after which the centrifuge tubes were placed in a high-speed centrifuge and spun at 10,000 rpm for 10 min. The supernatant then was passed through a 0.22 μm syringe filter for subsequent LC-MS/MS analysis.

Shellfish samples were extracted as follows: 100 g segments of the entire soft tissue of shellfish were collected and homogenized using a T25 Ultra Turrax homogenizer (IKA Works, Wilmington, NC, USA). Analyses of PSTs were conducted following published protocols [66,67]. Precise portions of 5 g (±0.05 g) of mixed tissue were extracted twice using a 1% aqueous acetic acid solution in a boiling water bath for 5 min. The combined supernatants were then transferred to a volumetric flask. A 1 mL portion of the extract was adjusted for pH using 5 μL of ammonia before vortex mixing, followed by centrifugation for 10 min at 13,000 rpm. The extract was then purified using solid-phase extraction. Supelco ENVI-Carb cartridges (250 mg, 3 mL, Sigma-Aldrich, St. Louis, MO, USA) were activated using sequential washes with acetonitrile, 20% acetonitrile aqueous solution (containing 1% acetic acid), and 0.1% ammonia. A volume of 500 μL of supernatant was loaded into the cartridge, and the cartridge was subsequently eluted with 700 μL of ultrapure water into a waste solution. Finally, 1 mL of 75% acetonitrile solution (containing 0.25% formic acid) was used for elution. All samples were centrifuged again for 5 min at 13,000 rpm, and the supernatant was stored at −80 °C for subsequent LC-MS/MS analysis.

### 4.4. LC-MS/MS Analysis

The PSTs were analyzed using a high-performance liquid chromatography system (Shimadzu Prominence LC-20ADXR, Shimadzu, Tokyo, Japan) in conjunction with a hybrid triple quadrupole linear ion trap mass spectrometer (5500 QTRAP LC-MS/MS system, AB Sciex Instruments, Foster City, CA, USA) equipped with a Turbo V source and an electrospray ionization probe. PSTs were screened in a hydrophilic column (TSK-Amide-80, 150 mm × 2.0 mm, 5 μm, Tosoh Bioscience GmbH, Griesheim, Germany) at 40 °C with a flow rate of 0.4 mL min^−1^ for the liquid chromatographic separation of PSTs. The instrument settings were as follows: ion spray voltage (positive mode), 5500 V; ion spray voltage (negative mode), 4500 V; curtain gas, 20 psi; GS1 and GS2, 50 psi; ion source temperature, 550 °C; and collision activation dissociation, medium. Nitrogen was used as both the nebulizer and collision gas in both ionization modes. The analysis was conducted using the multiple reaction monitoring mode. The LC parameters were based on the published studies [27]. Mobile phases A and B consisted of water and a solution containing 95% acetonitrile in water, respectively. Both mobile phases contained 2 mM ammonium formate and 50 mM formic acid. The elution gradient was as follows: the proportion of mobile phase B was 80% at the start and was held for 3 min, then linearly changed to 40% of mobile phase B within 5 min and then held for 2 min. Subsequently, it returned to 80% of mobile phase B within 1 min and was held for 2 min.

### 4.5. Statistical Analysis

All of the toxin composition and concentration data in the samples were analyzed using SPSS v. 26 (IBM Corporation, Armonk, NY, USA). *p* < 0.05 was considered to be statistically significant. The limit of detection (LOD) for analytes was determined based on a signal-to-noise ratio of 3:1, while the limit of quantification (LOQ) was ascertained with a signal-to-noise ratio of 10:1; when the toxin profile of a sample was higher than the LOD, it was regarded as a positive detection, and vice versa for non-detection. These results suggest that the PST-producing algae in the coastal waters of Changdao Island may belong to the *Alexandrium* spp. The toxicity of the various PSTs was converted according to their concentrations, and these toxicity equivalent factors were referenced to the EFSA standard [18]. The descriptive statistics and associated graphics were generated using Origin 2023.

## 5. Conclusions

This study investigated the potential sources of PSTs and the environmental factors influencing the distribution and number of toxin-producing algae in the coastal waters around Changdao Island. The results showed a widespread presence of PSTs in both plankton concentrates and shellfish samples, with the highest toxicities observed in September. The primary toxic algal genus identified was *Alexandrium*, potentially including species such as *A. hiranoi*/*pseudogonyaulax*, *A. pacificum*/*tamarense*, and *A. leei*. Future research should focus on identifying the specific species responsible for PST production and developing effective strategies to mitigate the risks associated with PST contamination.

## Figures and Tables

**Table 1:** The nine OTUs assigned to the various *Alexandrium* spp. observed.

OTUs	Species Assigned	Query Cover	Percent Identity
OTU1503	*A. pacificum/tamarense*	100%	98%
OTU48	*A. affine*	100%	98%
OTU349	*A. fraterculus*	100%	95%
OTU2155	*A. fraterculus*	100%	93%
OTU491	*A. hiranoi/pseudogonyaulax*	100%	100%
OTU725	*A. ostenfeldii*	100%	92%
OTU382	*A. leei*	100%	100%
OTU677	*A. leei*	100%	99%
OTU2136	*A. fraterculus*	100%	93%

## Data Availability

Data will be made available on request.

## Supplementary Materials

The following supporting information can be downloaded at https://www.mdpi.com/article/10.3390/md23050217/s1, Table S1: Monthly variations of hydrological and meteorological parameters and relative abundance of *Alexandrium* spp.
