# Supplementary material for: Paralytic Shellfish Toxins in Coastal Waters of Changdao Island (China): Toxin Profiles, Potential Producers, and Environmental Conditions
Source: Mar Drugs. 2025 May 21;23(5):217. doi: 10.3390/md23050217 (PMC12113264; doi:10.3390/md23050217)
Supplement: Supplementary file 1 [file marinedrugs-23-00217-s001.zip › marinedrugs-3354546-supplementary.pdf]

Supplementary materials

Table S1. Monthly variations of hydrological and meteorological parameters and relative abundance of *Alexandrium* spp.

Month	Wind speed (m/s)	Atmospheric pressure (Mpa)	Turbidity (NYU)	Transparency (m)	Sampling depth (m)	Proportion of *Alexandrium*.spp in phytoplankton communities (%)
Apr.	4.00	101.81	5.61	2.0	0.5	0
May	5.12	100.66	5.03	2.0	0.5	0.004352
Jun.	2.20	100.25	6.10	2.0	0.5	0.008963
Jul.	2.70	100.67	3.99	2.0	0.5	1.61
Aug.	2.10	100.71	4.30	1.5	0.5	1.50
Sep.	3.60	101.80	4.47	2.8	0.5	1.95
Oct.	5.00	102.69	14.65	1.2	0.5	0.33
